# Peer Review of “Mobile App–Reported Use of Traditional Medicine for Maintenance of Health in India During the COVID-19 Pandemic: Cross-sectional Questionnaire Study”

**DOI:** 10.2196/29632

**Published:** 2021-05-07

**Authors:** Ojelanki Ngwenyama

**Affiliations:** 1 Institute for Innovation and Technology Management Ted Rogers School of Management Ryerson University Toronto, ON Canada

**Keywords:** AYUSH Sanjivani app, COVID-19, traditional medicine, Ayurveda, Siddha, Unani, homeopathy

*This is a peer-review report submitted for the paper "Mobile App–Reported Use of Traditional Medicine for Maintenance of Health in India During the COVID-19 Pandemic: Cross-sectional Questionnaire Study"*

## Round 1:

I would like to thank the authors for doing an investigation of how health apps might help individuals manage their personal health. However, this paper makes some claims that cannot be defended based on the research design [1]. For example, the authors state, “79.1% of the users responded that the practice of AYUSH measures gave an overall feeling of good health and improved immunity.” I have no problem with claims of “overall feelings of good health”, but claims of improved immunity to COVID-19 are misleading. There is no possibility for the subjects to claim “improved immunity.” This would have required clinical testing for antibodies to the SARS-CoV-2 virus.

I believe that the fundamental problem is with the study design, especially the questions the researchers asked concerning reasons for using the AYUSH Sanjivani app: “Helped in prevention from COVID-19” and “Reduced the symptoms while having COVID-19.” There is no scientific basis to ask such questions in the context of their study.

## References

[1] Srikanth N, Rana R, Singhal R, Jameela S, Singh R, Khanduri S, Tripathi A, GoeL S, Chhatre L, Chandra A, Rao BCS, Dhiman KS (2021). Mobile App–Reported Use of Traditional Medicine for Maintenance of Health in India During the COVID-19 Pandemic: Cross-sectional Questionnaire Study. JMIRxMed.

